# Symbiotic diazotrophs in response to yak grazing and Tibetan sheep grazing in Qinghai-Tibetan plateau grassland soils

**DOI:** 10.3389/fmicb.2023.1257521

**Published:** 2023-09-06

**Authors:** Shengnan Sun, Yi Zhao, Quanmin Dong, Xiaoxia Yang, Yuzhen Liu, Wentao Liu, Guang Shi, Wenting Liu, Chunping Zhang, Yang Yu

**Affiliations:** ^1^College of Animal Science and Technology, Yangzhou University, Yangzhou, China; ^2^State Key Laboratory of Plateau Ecology and Agriculture, Qinghai Academy of Animal and Veterinary Science, Qinghai University, Xining, China

**Keywords:** Tibetan plateau, grazing patterns, *nifH* gene, soil microbes, alpine grassland

## Abstract

Grazing by local livestock is the traditional human practice in Qinghai-Tibetan Plateau grassland, and moderate intensity grazing can maintain high productivity and diversity of alpine grassland. Grazing ecosystems are often nitrogen-limited, but N_2_-fixing communities in response to yak grazing and Tibetan sheep grazing in Qinghai-Tibetan Plateau grassland have remained underexplored. In this study, we applied quantitative PCR quantitation and MiSeq sequencing of *nifH* under yak grazing and Tibetan grazing through a manipulated grazing experiment on an alpine grassland. The results showed that the grazing treatments significantly increased the soil ammonium nitrogen (AN) and total phosphorus (TP), but reduced the diazotrophs abundance. Compared with no grazing treatment, the composition of diazotrophs could be maximally maintained when the ratio of yak and Tibetan sheep were 1:2. The foraging strategies of grazing livestock reduced the legumes biomass, and thus reduced the diazotrophs abundance. Data analysis suggested that the direct key factors in regulating diazotrophs are AN and TP, and the changes of these two soil chemical properties were affected by the dung and urine of herbivore assemblages. Overall, these results indicated that the mixed grazing with a ratio of yak to Tibetan sheep as 1:2 can stabilize the soil diazotrophsic community, suggesting that MG12 are more reasonable grazing regimes in this region.

## 1. Introduction

Grasslands cover approximately above 60% of Qinghai-Tibetan Plateau (QTP) (Dong et al., 2020), and grazing by local livestock is the traditional human practice in this area. Natural grasslands are an essential fodder resource for herbivores and prevent the consumption of supplementary feed, contributing to the sustainability of the grazing system (Batalla et al., 2015). Most grasslands are grazed mainly by domestic livestock, from low, moderate, to high grazing intensity (Li et al., 2018). Overgrazing is a key driver which alters plant communities and soil nutrient availability, reducing productivity and sustainability of several ecosystem, especially in historically grazed grasslands (McSherry and Ritchie, 2013; García-Baquero et al., 2020; Alday et al., 2021). Although short-term high grazing intensity may stimulate the increase of soil organic matter and diversity in forage species composition, overgrazing can result in soil-degradation and loss of the fertile topsoil (Abdalla et al., 2018). Therefore, optimized management practices are required to balance the grassland and the livestock (Dong et al., 2014).

Previous results showed that historical grazing had significant negative legacy effects on the plant biomass, and the strong bottom-up controls of resource addition on soil food webs are mediated by the legacy of grazing intensity (Wang B. et al., 2021). According to the moderate disturbance hypothesis and the grazing optimization hypothesis, moderate grazing intensity generally increases grassland primary productivity (McNaughton, 1993). The peaks of aboveground net primary production (ANPP) at a moderate grazing intensity were consistent with predictions of the grazing optimization hypothesis (Altesor et al., 2005). Therefore, an optimal grazing regimes can promote the regrowth of the grazed plants, and beneficial interactions among the plant, soil, microorganisms and livestock of the degraded grasslands (Dong et al., 2020).

Nitrogen (N) availability predominantly limits grassland ecosystem primary productivity, which is an important factor in affecting grazing practice. Many microbially driven processes in soils can be impacted by land management practices and changes in the plant community (Lindsay et al., 2010). Most of the N_2_-fixing microorganisms carry *nif*H, which encodes one of the components of nitrogenase (Wang Y. et al., 2021). The effects of grazing practice on the abundance of functional genes involved in soil N cycling have been documented in recent years (Ding et al., 2014; Song et al., 2019). Diazotrophs are highly diverse in phylogeny and in a wide distribution in the QTP, the abundance, Shannon diversity, and community composition of soil diazotrophs were significantly correlated with soil moisture (Che et al., 2018), while another study showed that N-fixing communities (*nif*H) were most affected by the soil C:N ratio (Singh et al., 2011). Moreover, the variation in diazotroph community composition has a greater impact on N-fixation rates than did soil characteristics (Hsu and Buckley, 2009), it is inconsistent with a research in a tallgrass prairie used primarily for cattle grazing and agriculture, where they found that abundance of *nif*H genes was not significantly correlated with N2-fixation rates (Caton et al., 2018). In addition, there are weakly correlations between grazing and abundances of N functional genes in a grassy woodlands, frequent livestock grazing could lead to a reduction in the biological capacity for nitrogen fixation (Lindsay et al., 2010).

Most researchers had focused on the grazing intensity (McSherry and Ritchie, 2013) and grazing enclosure (grazing or non-grazing) experiment (Ding et al., 2014). However, there are many herbivores, such as yak and Tibetan sheep which as the dominant livestock play a crucial role in alpine grassland ecosystem functions on the QTP (Dong et al., 2014; Yang et al., 2019). Therefore, understanding how the ubiquitous symbiotic diazotrophs are affected by livestock species, especially mixing livestock species grazing patterns may have implications for the optimum model of grazing and sustainable development in an alpine pastoral region. In order to fill this knowledge gap, we collected soil samples from QTP with the mixing livestock species grazing history to assess: (a) How different grazing patterns affect the diazotrophs; and (b) What are the key factors affecting diazotrophs. We hypothesize that the above ground biomass and soil nutrients are affected by the foraging strategies of different livestock. To test this hypothesis, we investigated the abundance, diversity and community composition of diazotrophs under moderate grazing intensities, and examine the effects of yak grazing, sheep grazing and the mixing ratio of yak to sheep on the community composition of diazotrophs.

## 2. Materials and methods

### 2.1. Study sites and experimental design

This research was conducted at the town of Xihai, Haiyan Country (36°44′-37°39′ N, 100°23′-101°20′ E), Qinghai Province, China, situated in the Qinghai Lake basin. With an average elevation of 3,100 m, this region has a typical plateau continental climate. The mean annual temperature and precipitation were 1.4°C and 330–370 mm, respectively. Specifically, the non-growing season in this region is from October to April and is long and cold, with the average temperature of the coldest month being −24.8°C. The growing season is from May to September, with an average temperature of 12.5°C in the hottest months. Moreover, the precipitation also mainly concentrated in the growing season. The soil is a clay loam and the plant community is dominated by *Kobresia humilis* (C. A. Mey. ex Trautv.) Sergiev, *Leymus secalinus* (Georgi)Tzvel., *Elymus nutans* Griseb. Nachr. Ges. Wiss. Gott., *Carex aridula* V. Krecz. and *Potentilla acaulis* L. Sp. Pl.

We set up a grazing experiment with local livestock yak and Tibetan sheep started in 2014 with a completely randomized block design. It had been proved that moderate intensity grazing can maintain higher aboveground biomass and plant diversity (Gao and Carmel, 2020; Liu et al., 2023). Based on moderate intensity grazing, which was represented as the feed intake of livestock for above-ground biomass is about 50–55%, five grazing treatments including single grazing and mixed grazing were set up, which were: single grazing for yaks (YG), single grazing for Tibetan sheep (SG), mixed grazing for yaks and Tibetan sheep at the ratio of 1:2 (MG12), 1:4 (MG14), 1:6 (MG16), respectively, and a control treatment (CK, grazing exclusion) in three blocks, there were 18 plots in total. By adjusting the area of each grazing plot according to the livestock numbers, the grazing intensity of each plot was consistent. A detailed description of the experimental site and design can be found in our previous studies (Yang et al., 2019).

### 2.2. Sampling and measurement

In August 2019, four sites were randomly selected in each plot for plant community investigation and soil sampling. Aboveground biomass were harvested from a 0.5 m × 0.5 m quadrat in each sampling site. After plant community investigation, soil sampling was conducted in the same quadrat (Yang et al., 2019). Three random soil cores (0–10 cm, 10–20 cm and 20–30 cm) were taken in each quadrats. The samples from each quadrats were collected and mixed to form a composite sample by different depth. After careful removal of the surface litter, earthworms or bird droppings, each sample was mixed and divided into two parts. One part was air-dried for analysis of the soil physicochemical properties, and the other was stored in a freezer at −80°C and then sieved through a 2-mm sieve before analysis of the microbial communities.

The soil total carbon (TC), soil total nitrogen (TN), soil total phosphorus (TP), ammonium nitrogen (AN) and pH were measured following method of Huang (Huang et al., 2019).

### 2.3. Soil DNA extraction and real-time PCR

Microbial community genomic DNA was extracted using the E.Z.N.A.® soil DNA Kit (Omega Bio-tek, Norcross, GA, U.S.) according to manufacturer’s instructions. The DNA extract was checked on 1% agarose gel, and DNA concentration and purity were determined with NanoDrop 2000 UV–vis spectrophotometer (Thermo Scientific, Wilmington, United States).

The copies of *nifH* gene were quantified using ABI 7500 Real-Time PCR System (Applied Biosystems, Foster City, CA, USA). The quantification was conducted with universal primer sets for *nifH*, PolF: TGC GAY CCS AAR GCB GAC TC; PolR: ATS GCC ATC ATY TCR CCG GA (Poly et al., 2001; Che et al., 2018). The 10 μL reaction systems contained: 4.4 μL of SYBR Green Mix, 0.3 μL of forward primer (20 μmol L^−1^), 0.3 μL of reverse primer (20 μmol L^−1^) and 5 μL of template DNA. The standard curve was constructed using plasmids inserted with the *nifH* gene fragment. The PCR runs started with an initial denaturation at 95°C for 10 min, followed by 40 cycles of 10 s at 95°C, 34 s at 60°C, 15 s at 95°C, 60 s at 60°C and 1 cycle of 30 s at 95°C, 15 s at 60°C. The specificities of PCR products were checked by melting curve analysis.

### 2.4. MiSeq sequencing and bioinformatics analyses

The PCR amplifications of *nifH* gene was conducted with barcoded universal primer set (F: 5’-AAAGGYGGWATCGGYAARTCCACCAC-3′; R: 5’-TTGTTSGCSGCRTACATSGCCATCAT-3′). Purified amplicons were pooled in equimolar and paired-end sequenced on an Illumina MiSeq PE300 platform (Illumina, San Diego, United States) according to the standard protocols by Majorbio Bio-Pharm Technology Co. Ltd. (Shanghai, China). The raw *nifH* gene sequencing reads were demultiplexed, quality-filtered by fastp version 0.20.0 and merged by FLASH version 1.2.7 (Magoč and Salzberg, 2011). Operational taxonomic units (OTUs) with 97% similarity cutoff were clustered using UPARSE version 7.1, and chimeric sequences were identified and removed (Edgar, 2013). The taxonomy of each OTU representative sequence was analyzed by RDP Classifier version 2.2 against the 16S rRNA database using confidence threshold of 0.7 (Wang et al., 2007).

In total, 898,086 *nifH* gene sequences were obtained for the 54 soil samples, and the total sequence number was reduced to 859,258 after the quality filtering. The qualified sequence number of each sample ranged from 9,808 to 24,478, and thus the sequence number of each sample was rarefied to 9,808 for further analysis. With an identify cutoff of 97%, we obtained 2,421 OTUs. More details of the taxonomic assignment for *nif*H OTUs was conducted similarly as described by (Che et al., 2018). The data were analyzed on the online platform of Majorbio Cloud Platform.^1^

### 2.5. Statistical analyses

One-way ANOVA was used to analyze the effects of grazing on soil properties, abundance of *nif*H genes and OTU numbers, followed by Duncan’s new multiple range. Data were tested for normality and homogeneity of variance before analysis using the Shapiro–Wilk test and Levene’s Test, respectively. Two-way ANOVA was performed testing the main and interactive effects of grazing and soil depth on soil properties. Mantel tests were performed to assess correlations between diazotrophic taxa and experimental factors. These analyses were performed with the IBM Statistical Package, SPSS version 25.0 (IBM, Armonk, NY, United States), and the histogram plots were plotted by Sigma plot software. Linear regression analyses and structural equation model (SEM) were performed to test the relationships between the OTU numbers and environmental factors using the ‘ggplot2’ and ‘piecewiseSEM’packages in R version 4.1.0.

## 3. Results

### 3.1. Soil physiochemical properties with soil depth

Soil properties are shown in Table 1. A two-way ANOVA showed that the grazing treatments significantly affected all soil property parameters (*p*<0.01), and soil depth effect (0–10 cm, 10–20 cm and 20–30 cm) also influenced these parameters (*p*<0.01). Grazing treatments and soil depth had an interaction effects on soil ammonium nitrogen content (AN, *p*<0.01) (Supplementary Table S1). Compared with the no grazing CK, the YG and MG14 treatments significantly reduced the soil pH at 0–10 cm and 10–20 cm, respectively (*p*<0.05). Soil total carbon (TC) decreased significantly with the increase of soil layer in MG16 treatment (*p*<0.05). Soil total nitrogen (TN) under grazing treatment did not change significantly in soil layers 0–10 (*p*>0.05), but with the increase of soil layer, soil total nitrogen decreased significantly (*p*<0.05). In soil layers 10–20 and 20–30, soil total nitrogen under SG treatment was the highest, and soil total nitrogen under MG16 treatment was the lowest (Table 1). The grazing treatments significantly increased the soil AN (*p*<0.05), especially in the SG and YG treatments, and the AN are higher in the grazing alone treatment than that in the mixed grazing treatment. Moreover, compared to the CK, the grazing treatments also significantly increased the soil total phosphorus (TP, *p*<0.05), especially in the SG and YG treatment in soil depth of 0–10 cm (Table 1). In addition, the N/P ratio was lowest in SG treatment, while highest in MG12 treatment (p<0.05).

**Table 1:** Soil properties in different grazing treatments with soil depth.

Soil properties	Soil depth	CK	SG	YG	MG12	MG14	MG16
pH	0–10	7.84 ± 0.06a A	7.39 ± 0.22ab A	7.18 ± 0.22b B	7.44 ± 0.18ab A	7.08 ± 0.08b B	7.32 ± 0.18ab B
	10–20	7.97 ± 0.04a A	7.63 ± 0.22ab A	7.38 ± 0.05b AB	7.88 ± 0.08ab A	7.61 ± 0.24ab AB	7.87 ± 0.16ab A
	20–30	7.97 ± 0.09a A	7.74 ± 0.07a A	7.9 ± 0.22a A	7.83 ± 0.16a A	7.84 ± 0.15a A	8.16 ± 0.08a A
TC	0–10	37.85 ± 1.66a A	40.64 ± 2.79a A	41.76 ± 0.47a A	41.88 ± 0.75a A	41.81 ± 0.75a A	40.77 ± 0.31a A
	10–20	36.81 ± 1.59a A	39.64 ± 1.31a A	41.3 ± 1.53a A	38.94 ± 1.96a A	39.87 ± 0.68a A	37.72 ± 0.41a B
	20–30	36.4 ± 2.61ab A	38.12 ± 1.48ab A	37.46 ± 1.88ab A	37.15 ± 1.7ab A	40.67 ± 1.33a A	33.09 ± 0.39b C
TN	0–10	3.25 ± 0.13a A	3.49 ± 0.15a A	3.53 ± 0.1a A	3.54 ± 0.06a A	3.56 ± 0.11a A	3.38 ± 0.1a A
	10–20	2.79 ± 0.11ab A	3.17 ± 0.11a A	2.95 ± 0.22ab A	2.85 ± 0.19ab B	2.98 ± 0.09ab B	2.54 ± 0.17b B
	20–30	2.05 ± 0.17ab B	2.56 ± 0.06a B	2.03 ± 0.27ab B	1.98 ± 0.28ab C	2.43 ± 0.2ab C	1.75 ± 0.2b C
AN	0–10	0.91 ± 0.22c A	18.2 ± 1.04a A	15.87 ± 1.36a A	9.71 ± 0.62b A	9.86 ± 0.18b A	9.46 ± 1.34b A
	10–20	0.53 ± 0.06c AB	15.72 ± 1.69a A	13.28 ± 1.34a AB	5.78 ± 0.86b B	6.03 ± 0.28b B	5.28 ± 0.52b B
	20–30	0.28 ± 0.18d B	6.05 ± 0.83b B	10.24 ± 0.45a B	4.18 ± 0.49c B	4.74 ± 0.42bc C	4.97 ± 0.38bc B
TP	0–10	0.48 ± 0.01b A	0.55 ± 0.02a C	0.54 ± 0.01a A	0.51 ± 0.02ab A	0.54 ± 0.02a A	0.52 ± 0.01ab A
	10–20	0.43 ± 0.03b AB	0.52 ± 0.05ab A	0.49 ± 0.00ab B	0.51 ± 0.01ab A	0.58 ± 0.04a A	0.52 ± 0.02ab A
	20–30	0.39 ± 0.01c B	0.54 ± 0.01a A	0.46 ± 0.02b B	0.49 ± 0.03ab A	0.49 ± 0.03ab A	0.5 ± 0.03ab A
N/P	0–10	6.8 ± 0.12ab A	6.36 ± 0.13b A	6.47 ± 0.15ab A	6.97 ± 0.08a A	6.61 ± 0.02ab A	6.5 ± 0.31ab A
	10–20	6.47 ± 0.18a AB	6.28 ± 0.71a A	6.00 ± 0.43ab A	5.56 ± 0.26ab B	5.19 ± 0.32ab B	4.89 ± 0.22b B
	20–30	5.27 ± 0.59a B	4.77 ± 0.17a B	4.37 ± 0.46ab B	4.04 ± 0.37ab C	4.88 ± 0.27a B	3.46 ± 0.23b C
C/N	0–10	11.68 ± 0.75a A	11.64 ± 0.45a B	11.85 ± 0.29a B	11.83 ± 0.17a B	11.75 ± 0.23a B	12.09 ± 0.26a B
	10–20	13.26 ± 1.07ab A	12.51 ± 0.11b B	14.11 ± 0.6ab B	13.73 ± 0.46ab B	13.42 ± 0.40ab B	15.01 ± 0.94a AB
	20–30	18.20 ± 2.96a A	14.91 ± 0.26a A	19.13 ± 2.40a A	19.44 ± 2.29a A	16.96 ± 1.43a A	19.36 ± 2.00a A
C/P	0–10	79.44 ± 5.11a A	74.08 ± 3.24a A	76.62 ± 1.43a A	82.43 ± 1.26a A	77.72 ± 1.30a A	78.39 ± 2.10a A
	10–20	86.04 ± 9.21a A	78.71 ± 9.51a A	84.11 ± 3.07a A	76.24 ± 2.34a A	69.40 ± 3.15a A	73.11 ± 2.66a AB
	20–30	92.36 ± 3.15a A	71.15 ± 3.26bc A	81.57 ± 3.90ab A	76.76 ± 1.58bc A	82.44 ± 5.81ab A	66.02 ± 3.00c B

Lowercase letters in the table indicate significant differences between treatments in the same soil layer, and uppercase letters indicate significant differences between different soil layers of the same treatment.

### 3.2. The *nif*H gene abundance with soil depth

**Figure 1:**
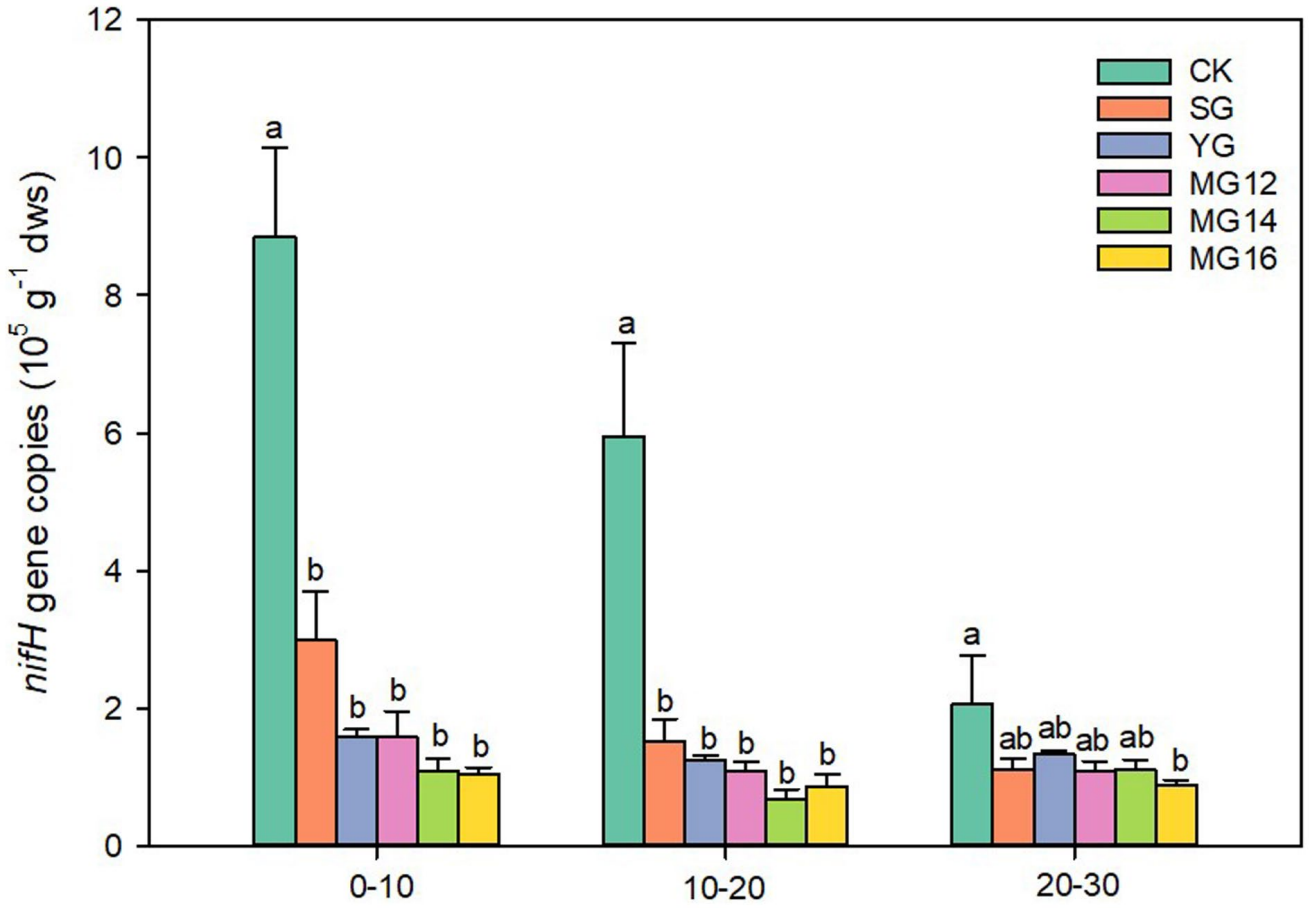
Effects of grazing patterns on the nifH gene abundance in different soil depth.

Compared with the CK, both grazing treatments significant decreased the *nif*H gene copy number in all soil depth (*p*<0.05) (Figure 1). The copy number of *nif*H gene in the CK treatment decreased significantly with the increase of soil layer (*p*<0.05), whereas no significant change was observed among the other grazing treatment (*p*>0.05). In the 0–10 cm from the soil surface, the average copy numbers of the six treatments ranged from 0.73 × 10^5^ to 9.19 × 10^5^ gene copies per g dry soil (Figure 1).

### 3.3. OTU richness and relative abundance of diazotrophic taxa

**Figure 2:**
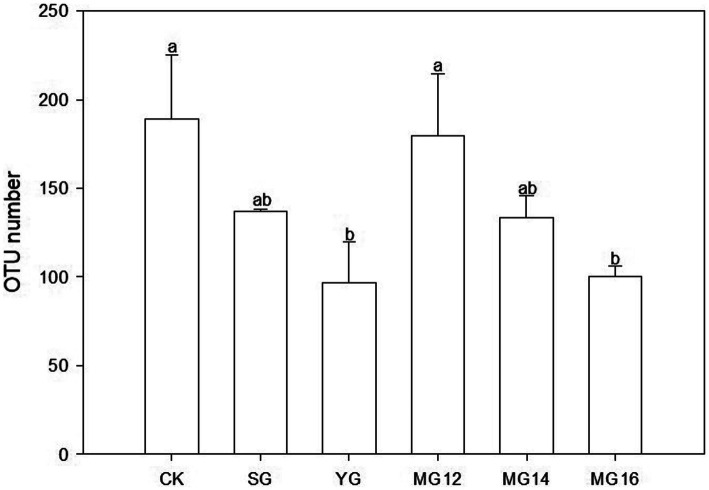
Effects of grazing patterns on the OTU numbers in 0–10 cm of soil surface.

**Figure 3:**
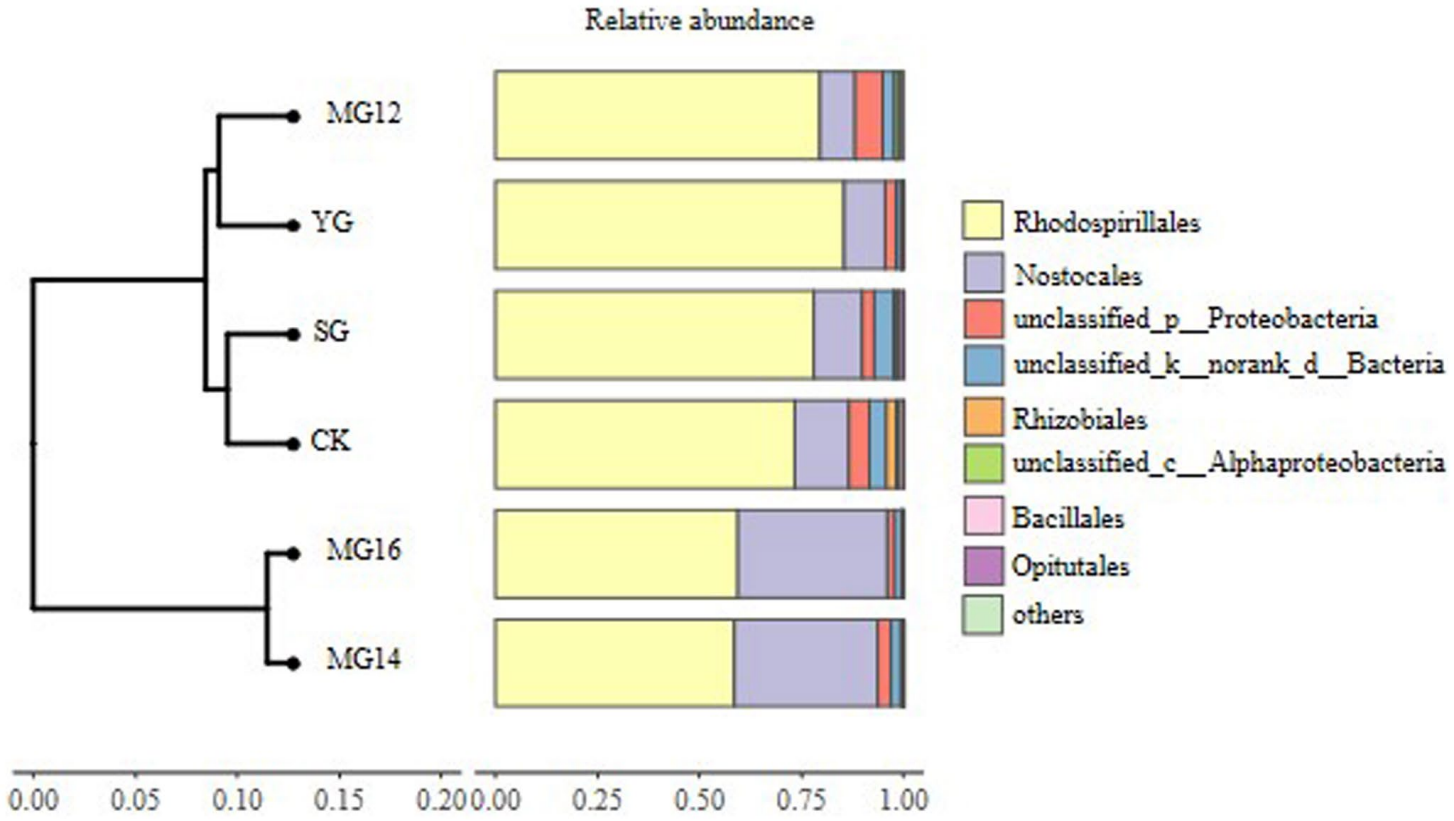
Relative abundances of diazotroph community response to different grazing patterns in the soil at the order level.

The CK and MG12 treatments had similar OTU numbers, and the YG and MG16 treatments had the lower the number of OTUs (*p*<0.05) (Figure 2). There were no significant differences among CK, SG, MG12 and MG14 (*p*>0.05) (Figure 2). OTUs were taxonomically classified into different order across all of the treatments. Figure 3 shows the relative abundances of the most abundant order in soils of the six grazing treatments. The order *Rhodospirillales* was the most abundant, containing 58–81% of the total *nifH* gene sequences in all of the soil samples. Different grazing treatments changed the relative abundances of these main order (Figure 3). Compared with CK, SG, YG and MG12 had similar dominant communities, while the relative abundances of *Rhodospirillales* decreased and *Nostocales* increased in the MG14 and MG16 treatments.

### 3.4. Correlation among diazotrophic community structure, soil variables and plant biomass

**Figure 4:**
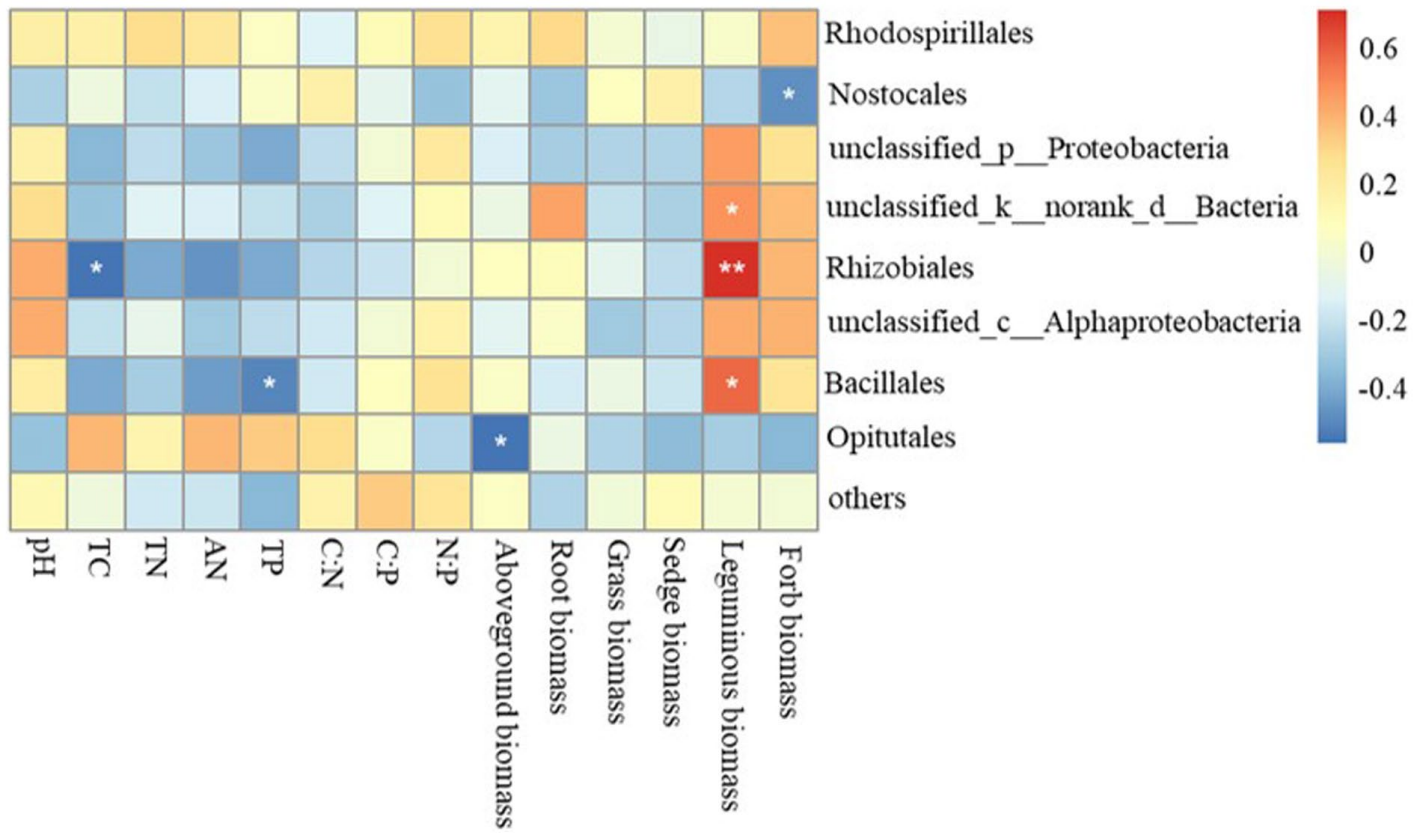
Relationships between variables related to soil diazotroph community, soil characteristics and plant functional community biomass. TC, soil total carbon; TN, soil total nitrogen; AN, soil ammonium nitrogen; TP, soil total phosphorus.

**Figure 5:**
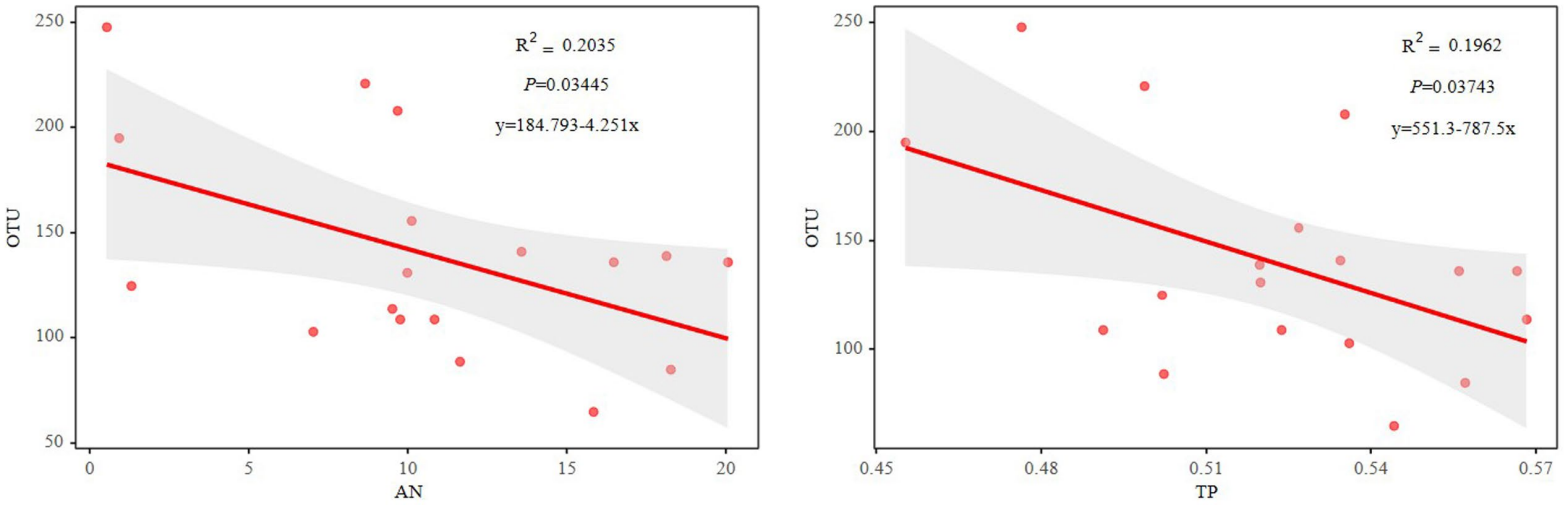
Relationships between the OTU numbers and soil AN or TP. AN, soil ammonium nitrogen; TP, soil total phosphorus.

The Mantel test revealed that the diazotrophic community structures in soil surface were closely correlated with multiple soil variables, such as pH, TC, AN and TP in the 0–10 cm of soil (p<0.05) (Table 2). then the *nifH* gene copies only had a negative correlation with TP in 10–20 cm and 20–30 cm of soil layers (p<0.05). whereas had no significant correlation with aboveground biomass and root biomass (p>0.05) (Table 2). However, specifically, *Rhizobiales* and *Bacillales* are closely related to leguminous biomass, and they are related to soil TC and TP, respectively. In addition, *Nostocales* is related to forb biomass, and *Opitutales* is related to aboveground biomass (Figure 4). Based on the linear regression analysis, there are negative correlations between OTUs number, AN and TP in the 0–10 cm of soil (*p*<0.05) (Figure 5).

**Table 2:** Correlations among the *nifH* gene copies, soil properties and plant community biomass.

	pH	TC	TN	AN	TP	C/N	C/P	N/P	Aboveground biomass	Root biomass
*nifH* gene copies in 0–10	0.602**	−0.664**	−0.417	−0.595**	−0.534*	0.111	−0.134	0.199	0.315	−0.227
*nifH* gene copies in 10–20	0.349	−0.414	0.021	−0.449	−0.502*	−0.067	0.262	0.460	0.333	0.092
*nifH* gene copies in 20–30	−0.046	−0.191	0.064	−0.325	−0.557*	−0.223	0.385	0.497*	0.300	0.186

Significant correlations are highlighted in bold font (*p* < 0.05).

**Figure 6:**
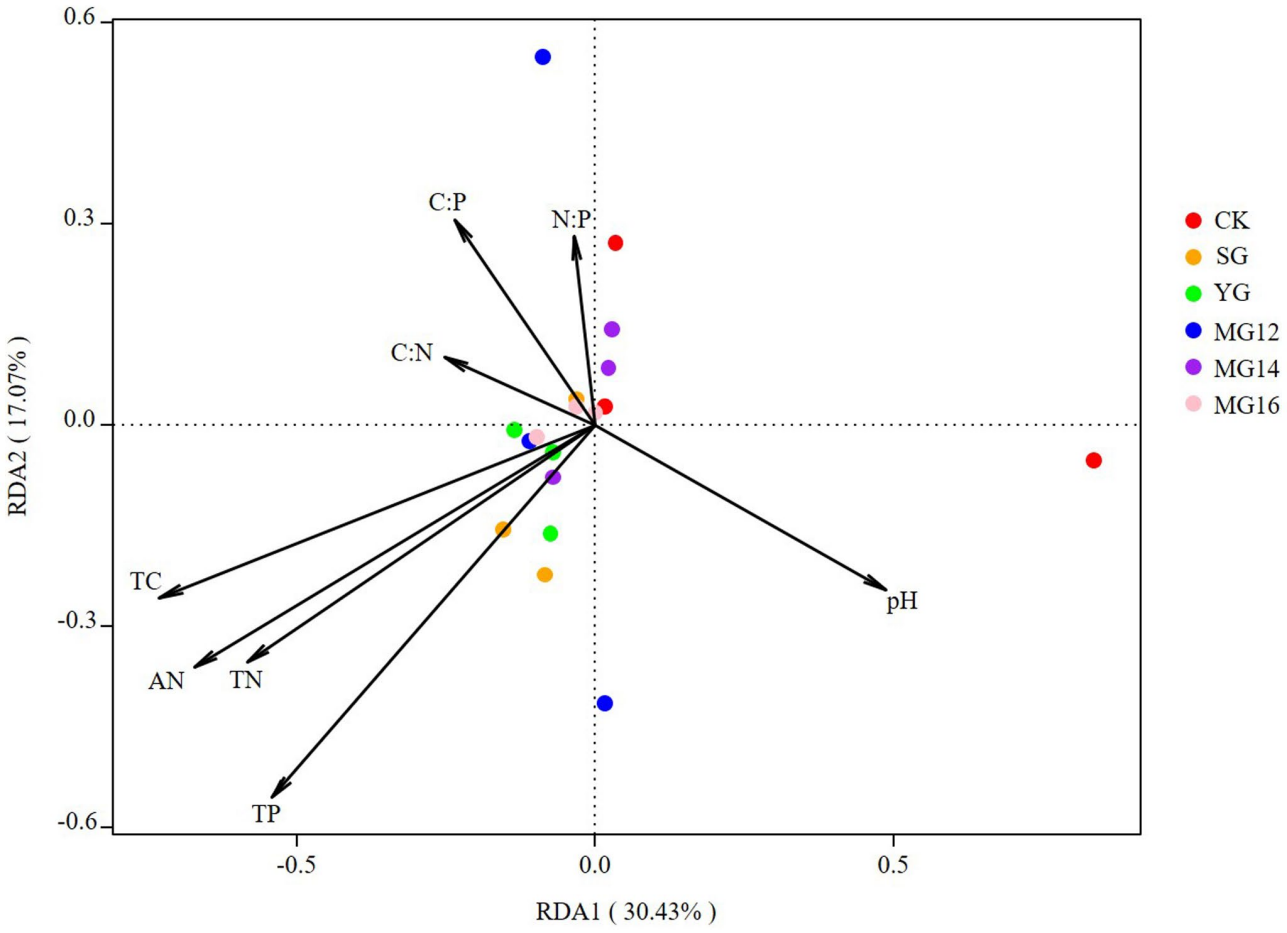
Relationships between soil properties and diazotroph community as shown by redundancy analysis (RDA).

**Figure 7:**
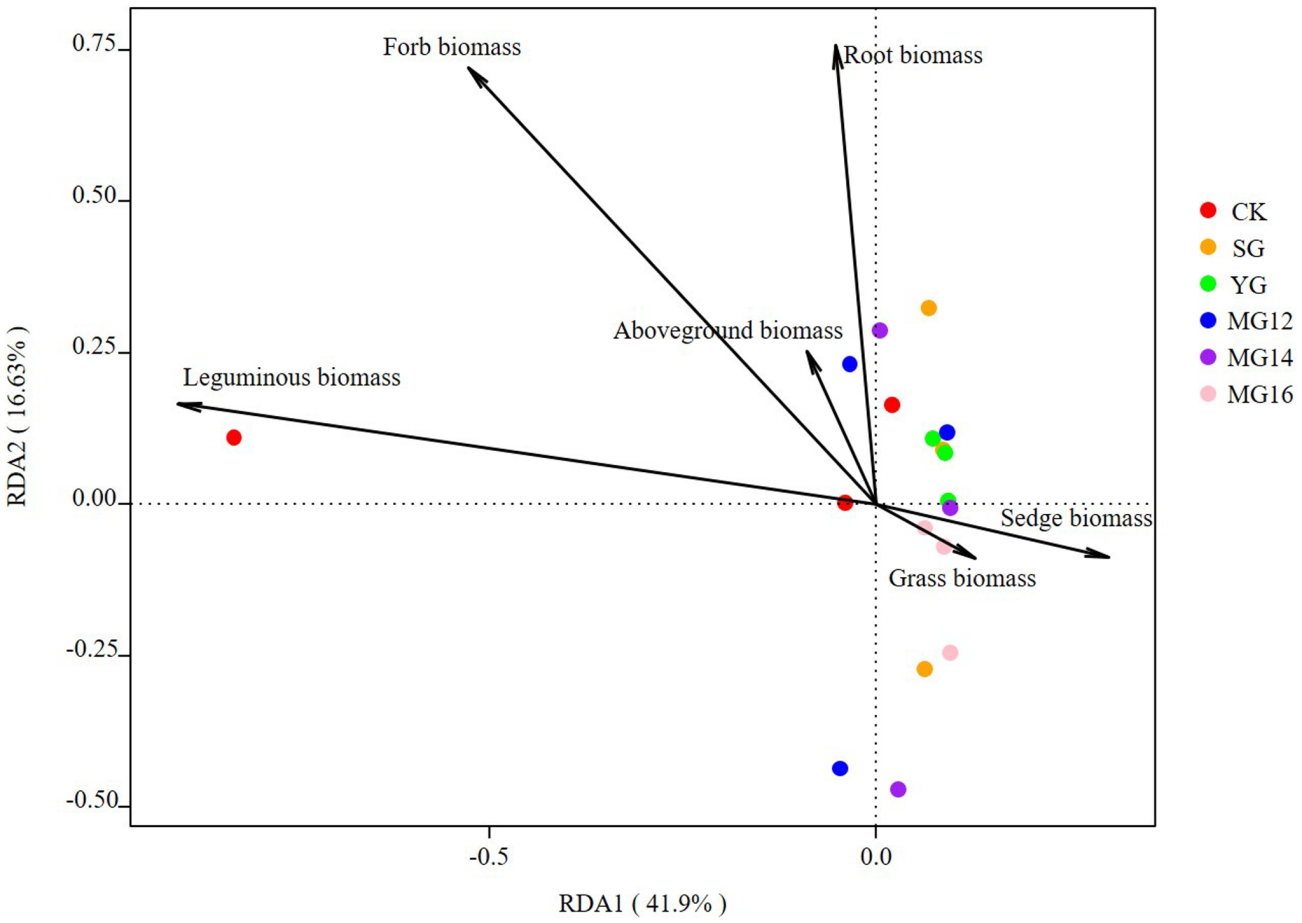
Relationships between plant functional community biomass and diazotroph community as shown by redundancy analysis (RDA).

The effects of soil properties and plant functional group on the structures of diazotrophic communities were further analyzed using the redundancy analysis, based on the selected soil variables, biomass of plant functional group and OTU composition. These soil variables explained 47.5% of the variation, and the first two axes explained 30.43 and 17.07% of the total variation. According to the vectors, the diazotrophic communities of both CK and grazing treatments were associated with higher TP and AN values (Figure 6). In addition, the diazotrophic communities were also associated with leguminous biomass and forbs biomass. These biomass of plant functional group explained 58.53% of the variation, and the first two axes explained 41.9 and 16.63% of the total variation (Figure 7).

### 3.5. Key factors driving changes in diazotrophic communities

**Figure 8:**
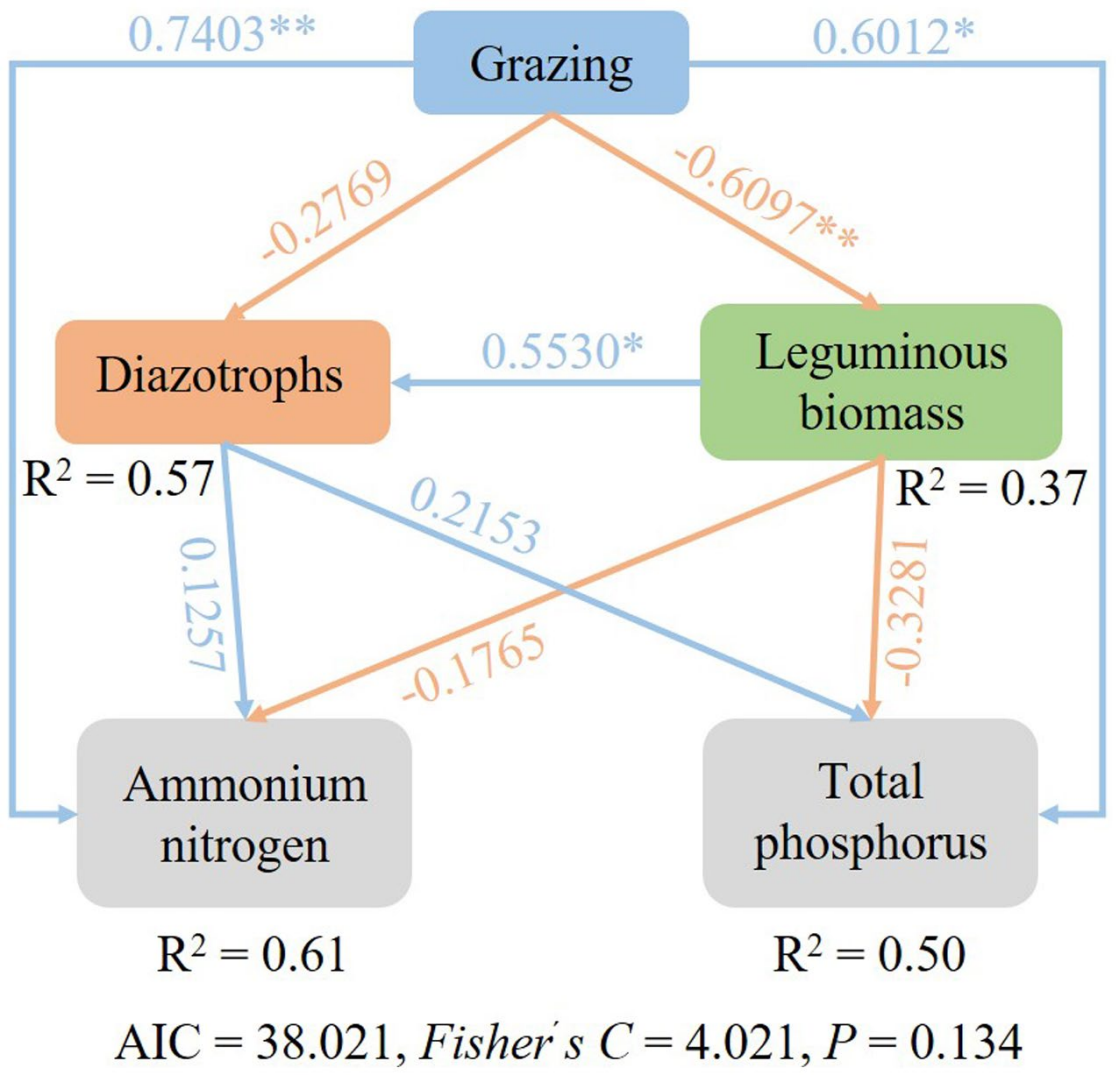
Structural equation model (SEM) describing the effects of grazing on soil diazotroph community.

SEM were used to identify the key drivers on diazotrophic communities. Path analysis indicated that grazing had an impact on diazotrophs through the direct effects on leguminous biomass, AN and TP content. Grazing reduced the biomass of legumes (Supplementary Figure S1), but increased AN and TP content, and legumes were positively correlated with nitrogen-fixing bacteria, so nitrogen-fixing bacteria also decreased under grazing treatment (Figure 8).

## 4. Discussions

### 4.1. Diazotrophic abundance

Many microbially driven processes in soils can be impacted by land management practices and changes in the plant community (Lindsay et al., 2010; Meyer et al., 2013). Previous studies had described the diversity, composition and structure of the diazotroph community under different systems (Song et al., 2019; Xiao et al., 2020a; Barros et al., 2021). However, little information is available about the changes of diazotrophic community in Qinghai-Tibetan Plateau grassland soils with different grazing patterns. Thus, this study evaluated the influence of grazing patterns on diazotrophic communities in Qinghai-Tibetan Plateau grassland under moderate intensity grazing. The decreased of *nifH* gene copies under all grazing treatments were consistent with previous reports (Yan et al., 2021). In the present study, compared with CK, both singly and mixed grazing treatment reduced the *nifH* gene copies. This is consistent with Lindsay’s findings, they found that there was a trend for higher abundance of *nifH* in sites where grazing was excluded (Lindsay et al., 2010). The significant negative relationship between *nifH* gene abundance and soil physicochemistry (TC, AN and TP) under grazing treatment, suggested that frequent livestock grazing could inhibit dinitrogenase reductase and lead to a reduction in the biological capacity for nitrogen fixation. A large proportion of the nitrogen in dung and urine from livestock can provide high nitrogen inputs in various forms, therefore can reduce the ecosystem dependence on free-living nitrogen fixing organisms (Singh et al., 2011).

### 4.2. Compositions and structures of the diazotrophic communities

With regard to the effects of grazing patterns on the diazotrophic community composition, a previous study showed that intensive grazing may increased the relative abundances of *Proteobacteria*, *Bacteroidetes* and *Firmicutes* (Zhang et al., 2023), whereas another study found that grazing exclusion increased the relative abundance of these bacteria (Wang Z. et al., 2021). In our study, YG and MG16 significant reduced the OTUs number of diazotrophs, and the dominant diazotrophic communities belong to *Proteobacteria*, *Firmicutes*, *Rhizobiales*. The high proportions of *Proteobacteria* in CK and MG12 treatment indicate the mix grazing patterns with ratios of yak to sheep as 1:2 is closed to the natural grassland which without grazing in regulating soil microbial community. Meanwhile, the dominant bacteria had significant correlation with legume biomass suggested the abundance of nitrogen-fixing bacteria in soil is influenced by the foraging strategies of different livestock. Among the *Proteobacteria* and *Firmicutes* phylum, taxa such as *Rhizobiales* and *Bacillales* species are well known for forming root nodules with legumes and conducting symbiotic nitrogen fixation, and varying significantly in abundance among the types of land use (Yan et al., 2021).

### 4.3. Diazotrophs in response to yak grazing and Tibetan sheep grazing

Both of the *nifH* gene copies and OTUs had negative correlation with soil TP. Although diazotrophic communities were sensitive to phosphorus, a study in karst ecosystems reported that soil P availability played an important role in regulating N_2_ fixation by increasing diazotroph diversity (Xiao et al., 2020b), it was inconsistent with our results. Our preliminary work found that the response of soil TP showed a strong dependence on livestock species (Yang et al., 2019), the responses of TP to grazing were significantly positively related to the proportion of sheep in the mixed livestock group. This suggests that changes in N_2_-fixing communities may also be related to the proportion of sheep in mix grazing. In addition, grazing can also increase soil AN content due to the feces and urine of small herbivores have a relative high N content (Liu et al., 2023). Previous studies indicated that high available nitrogen content had a negative effect on soil diazotrophs as the nitrogenase enzyme is sensitive to ammonia (Che et al., 2018; Han et al., 2019). Moreover, the increased content of TP and AN enriched soil available nutrients and reduced the dependence of plants on nitrogen-fixing bacteria. Consequently, this could explain why *nifH* gene copies decreased in grazing treatments. Livestock grazing in grassy woodlands could potentially alter the nitrogen cycle (Lindsay et al., 2010), and grazing has been found to enhance the activity of soil nitrifying and denitrifying bacteria (Patra et al., 2005). However, herbivory can impose strong limits on diazotroph plant host abundance (Ritchie et al., 1998). Meanwhile, pasture management may affect the bacterial community through change the botanical composition and pasture productivity (Wakelin et al., 2009), and the grass roots can harbor abundant endophytic N_2_-fixing microbes (Ritchie and Raina, 2016).

### 4.4. Key factors driving diazotroph community

The type of land management and land use intensity has been identified as a major driver for microbial performance in soil (Meyer et al., 2013). In this study, grazing treatment had a significantly effects on soil AN. The soil AN and *nifH* were negatively correlated in different grazing patterns, suggesting that aboveground herbivory reduces the capacity for belowground nitrogen fixation (Lindsay et al., 2010). The composition and activities of diazotrophic communities are essential for the functioning of the soil nutrient cycles (Hsu and Buckley, 2009). Due to plants and soils can form a complex mutual feedback relationship, the root biomass and aboveground biomass of legume and herb also play an important role in regulating the diazotrophic communities. Plant communities and soil variables can potentially produce an evident interaction in their influence on the soil organic carbon and nitrogen contents (Yuan and Jiang, 2021). As the most important and common land use of grasslands, livestock grazing and especially over-grazing can alter the composition and productivity of the plant community and can accelerate the loss of soil fertility and the depletion of other resources (Wang B. et al., 2021). In the present study, the herbivore assemblage did not change the diversity of the N_2_-fixing communities, but significantly reduced their abundance. Moreover, MG12 and CK had similar OTUs of diazotrophs, which revealed that the composition of N_2_-fixing microorganisms could be maximally maintained when yak and Tibetan sheep were 1:2. Based on SEM, the effects of grazing on diazotrophs were achieved by changing leguminous biomass, soil AN and soil TP, respectively.

## 5. Conclusion

Following the grazing treatment in the alpine meadow, variations of plant communities and soil chemistries influenced the composition of N_2_-fixing microbial communities. The results of this study showed that although the grazing treatments significantly reduced the diazotrophs abundance, the OTUs riches of MG12 were closed to grazing exclusion, this suggested that mixing grazing in the ratio of yak to Tibetan sheep 1:2 maintained a relatively stable community structure of diazotrophic communities. Moreover, the effects of grazing on diazotrophs can be seen in two ways: foraging behavior of livestock reduced the legume biomass and simultaneously reduced the abundance of diazotrophs which closely related to legume; the other way is that the feces and urine of livestock increased the soil ammonium nitrogen and total phosphorus content, thereby inhibiting the abundance of diazotrophs. Based on previous studies in this platform and from a management perspective, MG12 can improve above-ground net primary productivity and is less damaging to diazotrophs. Therefore, under the background of moderate intensity grazing, mixed grazing of yak and Tibetan sheep at a ratio of 1:2 is an optimal choice for the QTP.

## Data availability statement

The datasets presented in this study can be found in online repositories. The names of the repository/repositories and accession number(s) can be found at: NCBI: SAMN36704862 – SAMN36704915.

## Author contributions

SS: Data curation, Funding acquisition, Writing – original draft, YZ: Formal analysis, Writing – original draft. QD: Resources, Writing – review & editing. XY: Methodology, Resources, Writing – review & editing. YL: Data curation, Writing – original draft. WentaL: Investigation, Methodology, Writing – original draft. GS: Investigation, Writing – original draft. WentiL: Investigation, Writing – original draft. CZ: Investigation, Writing – review & editing. YY: Investigation, Writing – original draft.

## Funding

The author(s) declare financial support was received for the research, authorship, and/or publication of this article. This work was supported by the Open Project of State Key Laboratory of Plateau Ecology and Agriculture, Qinghai University (2019-KF-004), and the Science and Technology Basic Condition Platform of Qinghai Province Science and Technology Department (2020-ZJ-T07).

## Conflict of interest

The authors declare that the research was conducted in the absence of any commercial or financial relationships that could be construed as a potential conflict of interest.

## Publisher’s note

All claims expressed in this article are solely those of the authors and do not necessarily represent those of their affiliated organizations, or those of the publisher, the editors and the reviewers. Any product that may be evaluated in this article, or claim that may be made by its manufacturer, is not guaranteed or endorsed by the publisher.

## Footnotes

^1^ www.majorbio.com
