# Supplementary material for: Symbiotic diazotrophs in response to yak grazing and Tibetan sheep grazing in Qinghai-Tibetan plateau grassland soils
Source: Front Microbiol. 2023 Sep 6;14:1257521. doi: 10.3389/fmicb.2023.1257521 (PMC10511875; doi:10.3389/fmicb.2023.1257521)
Supplement: Supplementary file 1 [file Data_Sheet_1.pdf]

Table S1 The interaction effect of Treatment (T) and Soil Depth (D) on soil properties.

Soil properties	T		D		T × D	
	F	*p*	F	*p*	F	*p*
pH	**3.747**	**0.008**	**18.581**	**0.000**	1.101	0.388
TC	**3.225**	**0.017**	**8.848**	**0.001**	0.848	0.588
TN	**3.967**	**0.006**	**99.056**	**0.000**	0.824	0.609
AN	**99.972**	**0.000**	**67.915**	**0.000**	**6.901**	**0.000**
TP	**8.415**	**0.000**	**6.039**	**0.005**	1.168	0.343
C/N	1.426	0.238	**36.817**	**0.000**	0.476	0.894
C/P	**3.697**	**0.008**	0.016	0.984	1.768	0.103
N/P	**4.488**	**0.003**	**65.248**	**0.000**	1.750	0.107

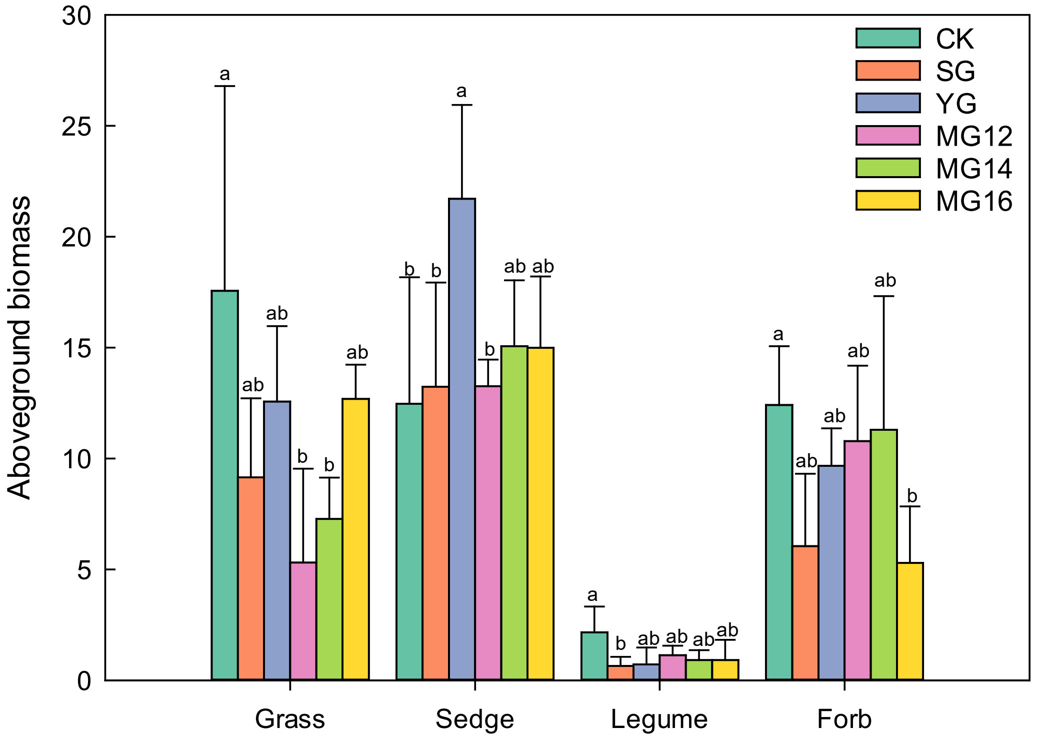

FIGURE S1 Effects of grazing patterns on the aboveground biomass of functional groups
